# Interpreting local visual features as a global shape requires awareness

**DOI:** 10.1098/rspb.2010.1909

**Published:** 2010-12-08

**Authors:** D. Samuel Schwarzkopf, Geraint Rees

**Affiliations:** 1 UCL Institute of Cognitive Neuroscience, 17 Queen Square, London WC1N 3AR, UK; 2 Wellcome Trust Centre for Neuroimaging at UCL, 12 Queen Square, London WC1N 3BG, UK

**Keywords:** awareness, integration, orientation, invisible

## Abstract

How the brain constructs a coherent representation of the environment from noisy visual input remains poorly understood. Here, we explored whether awareness of the stimulus plays a role in the integration of local features into a representation of global shape. Participants were primed with a shape defined either by position or orientation cues, and performed a shape-discrimination task on a subsequently presented probe shape. Crucially, the probe could either be defined by the same or different cues as the prime, which allowed us to distinguish the effect of priming by local features and global shape. We found a robust priming benefit for visible primes, with response times being faster when the probe and prime were the same shape, regardless of the defining cue. However, rendering the prime invisible uncovered a dissociation: position-defined primes produced behavioural benefit only for probes of the same cue type. Surprisingly, orientation-defined primes afforded an enhancement only for probes of the opposite cue. In further experiments, we showed that the effect of priming was confined to retinotopic coordinates and that there was no priming effect by invisible orientation cues in an orientation-discrimination task. This explains the absence of priming by the same cue in our shape-discrimination task. In summary, our findings show that while in the absence of awareness orientation signals can recruit retinotopic circuits (e.g. intrinsic lateral connections), conscious processing is necessary to interpret local features as global shape.

## 1. Introduction

One of the key functions of the visual system is to construct a coherent representation of the environment. It must identify the local features belonging to the same object, such as edges, corners and surfaces, and bind them together into a global percept. The human brain accomplishes this task with astonishing ease, even under conditions of great ambiguity, when the object of interest is occluded by other surfaces and in the presence of distracting edges, any of which could be interpreted to also belong to the target object. The visual system is optimized to facilitate the extraction of meaningful information from such noisy input [1–3]. However, one question has not been explored in this context: to what extent does this process of perceptual integration depend on conscious awareness of the visual stimulus?

The answer to this question relates to another larger question: what is consciousness for? Previous research exploring the differences between conscious and unconscious processing suggested that a great deal of information about a stimulus is present in the visual system even when participants are unaware of it. For example, stimuli masked from awareness can produce afterimages and contextual illusions [4,5]. Brain-imaging experiments show that the primary visual cortex encodes the orientation of a stimulus even when it is rendered invisible by a mask [6]. Furthermore, extrastriate brain regions contain a representation of complex visual objects, such as faces and houses, in the absence of awareness [7–9]. Invisible images of fearful or surprised faces produce responses in the brain showing that not only visual information but also complex emotional content is conveyed by unconscious processing [10,11].

Nonetheless, the nature of an object representation under conditions when the object is not consciously perceived remains unclear. Recent studies suggest that while patterns of voxel activity in the ventral visual cortex can be used to discriminate responses to invisible faces and houses, these patterns may be different from those evoked by visible stimuli [8,9]. It has been proposed that the representation of invisible stimuli in intermediate visual areas is characterized by larger variability, but this interpretation is complicated by the presence of physical differences between the visible and invisible stimuli [9]. However, in line with this interpretation, psychophysical experiments also indicated that, even under conditions of equal stimulation during binocular rivalry, neuronal tuning widths are increased when a stimulus is suppressed from awareness [12].

**Figure 1.**
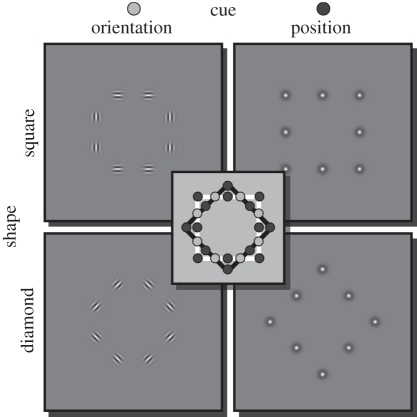
Shapes could be constructed by orientation or position cues. The shape was either a square or a diamond. At the intersections of the two shapes (in schematic, denoted by light grey circles), only the orientation of the element provides shape information. At the corners and middle of the sides (dark grey circles), orientation would be at best ambiguous and only position information is useful for shape discrimination.

One way in which conscious and unconscious visual processing may differ is in the extent to which local image attributes are integrated into global shape. Here, we aimed to address this question directly with a behavioural priming paradigm. Our stimulus design allowed us to disentangle the effects of local stimulus features compared with the representation of the global shape (figure 1). The stimuli could be defined either by the position of elements lacking any orientation information (experiment 1), or by only the orientation of local elements without any positional cues (experiment 2). Participants were asked to perform a simple shape-discrimination task. Under normal viewing conditions, we predicted that if a shape stimulus was preceded by a prime stimulus of the same shape, discrimination performance would improve, regardless of whether the prime was defined by positional or orientation cues. However, we reasoned that rendering the prime invisible would allow us to distinguish whether there was a representation of the global shape in the absence of awareness, or whether performance benefits were confined to local stimulus features. In a further experiment, we tested whether priming would be observed when the probe shape was presented at a smaller scale that would rule out purely retinotopic effects (experiment 3). Finally, we tested whether invisible priming was observed for discriminating the local orientation of elements without a global shape interpretation (experiment 4).

## 2. Material and methods

### (a) Experiments 1 and 2

#### (i) Stimuli

All stimuli comprised eight local elements, which were arranged in the shape of either a square or a diamond (i.e. a square rotated by 45°). Similar to a previous study [13], our stimulus design exploited unique locations on the shape (figure 1, centre): elements positioned at the intersections of the two shapes (white circles) must contain orientation cues to permit discrimination of the shapes. Conversely, at the corners and in the middle of each side of the squares (dark grey circles), the position of the elements is maximally informative about the shape, while orientation cues are ambiguous. Thus, shape stimuli could be defined by orientation cues or by position cues (see figure 1 and electronic supplementary material for details).

#### (ii) Procedure

**Figure 2.**
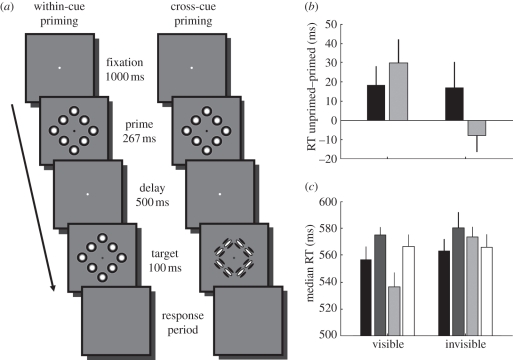
Position-defined priming in experiment 1. (*a*) Trial sequence. The prime was always defined by position cues. The probe could either also be defined by position cues (within-cue) or by orientation cues (cross-cue). Shown here are primed trials, in which the probe and prime are the same shape. In unprimed trials, the probe would be the opposite shape as the prime. (*b*) The priming effect (how much faster response times were for primed relative to unprimed trials) for the four priming conditions: visible and invisible primes, within-cue and cross-cue condition. (*c*) Median response time for every trial type. In both (*b*) and (*c*), data reflect mean across participants, and error bars denote ±1 s.e. of the mean (between-subject variance removed). RT, response time. (*b*) Black bars, within-cue priming; grey bars, cross-cue priming. (*c*) Within-cue: black bars, primed; dark grey bars, unprimed; cross-cue: light grey bars, primed; white bars, unprimed.

**Figure 3.**
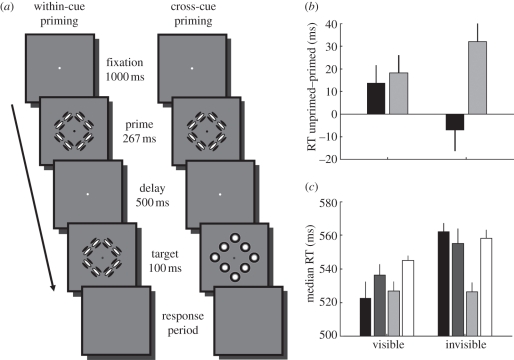
Orientation-defined primes in experiment 2. (*a*) Trial sequence. The prime was always defined by orientation cues. The probe could either also be defined by orientation cues (within-cue) or by position cues (cross-cue). (*b*) The priming effect. (*c*) Median response time for every trial type. All other details are identical to figure 2. (*b*) Black bars, within-cue priming; grey bars, cross-cue priming. (*c*) Within-cue: black bars, primed; dark grey bars, unprimed; cross-cue: light grey bars, primed; white bars, unprimed.

On each trial, participants were required to fixate on a small white dot in the centre of the screen for 1000 ms. Then, a prime shape was presented for 267 ms, followed by a 500 ms inter-stimulus interval. Subsequently, a probe shape, which could be one of the four possible stimulus shapes (figure 1), was presented for 100 ms, after which the screen went blank and participants were asked to respond whether the probe was a square or a diamond. The response was self-paced; however, participants were instructed to respond as quickly as possible. The fixation dot was present for the entire trial until the response period. However, as an aide to help participants know which stimulus to respond to, during the prime presentation the dot was black and during the probe presentation it was blue. The trial sequence of the two types of primed trials is shown in figures 2*a* and 3*a* (for experiments 1 and 2, respectively). After each trial, participants received feedback in the form of a small coloured circle (width: 0.85° of visual angle) presented at fixation for 100 ms (green, correct; red, incorrect).

The prime shapes were either always defined by position cues (experiment 1) or by orientation cues (experiment 2). Moreover, on half the trials, the prime was rendered invisible by counter-phase flickering of the stimulus contrast at 120 Hz. In half the trials, the prime and probe were the same shape (primed trials). Within-cue priming was assumed to occur if the prime and probe also shared the defining cue. Conversely, priming of the global shape representation must occur for cross-cue priming (i.e. when the defining cue differs between the two stimuli).

### (b) Experiment 3

#### (i) Stimuli

Stimuli were identical to those used in experiment 2, except that the probe stimulus was scaled to 62.5 per cent of the size of the prime (including the wavelength/standard deviations of the stimulus elements).

#### (ii) Procedure

Task and trial sequence were identical to experiment 2 except that all primes were defined by orientation cues.

### (c) Experiment 4

#### (i) Stimuli

Stimuli comprised eight elements, all of which were Gabor patches (i.e. orientation cues). The stimulus design ensured that the content of local information was comparable to the orientation-defined shape stimuli of the other experiments, while there was no global shape interpretation (see electronic supplementary material for details).

#### (ii) Procedure

The trial sequence was identical to the previous experiments except that, instead of discriminating the shape of the stimulus, participants were required to distinguish whether the orientations of the elements were cardinal or oblique. On primed trials, the prime and probe were identical, and in the unprimed trials, the orientation of the local elements was changed to the opposite stimulus (e.g. from cardinal to oblique). The position of elements was always identical in both intervals.

Further details about participants and methods used in all four experiments can be found in the electronic supplementary material.

## 3. Results

### (a) Experiment 1: position-defined primes

We used behavioural priming to test whether, in the absence of awareness, position cues can be grouped into a representation of global form. Participants reported whether the probe shape was a square or a diamond. On primed trials, the prime was the same shape as the probe. We reasoned that under these circumstances, behavioural shape-discrimination performance should be improved compared with unprimed trials in which the prime and probe were opposite shapes. Crucially, while the prime was always defined by position cues, the probe could be defined either by orientation cues or by position cues (figure 2*a*). If any priming benefit was observed in the latter type of trial, the *cross-cue priming* condition, this must tap into mechanisms integrating local stimulus features into a global shape percept. Conversely, in the *within-cue priming* condition, behavioural performance may be influenced by both the global shape representation and local features.

The shape-discrimination task was very easy, as reflected by very high accuracies in all participants (mean: 0.94, minimum: 0.81). Therefore, we did not expect to see any considerable priming effects on accuracy. Instead, we posited that priming would result in shorter response times, and in order to maximize this effect, we instructed participants to respond as quickly as possible. The strength of the priming effect was calculated by subtracting the median response time to unprimed trials by that to primed trials for each participant. Thus, a positive value indicates faster responses to primed trials. Because we were interested in whether global priming would be observed even when the prime was rendered invisible, we limited our main analyses to only those participants who showed cross-cue priming for *visible* primes. Seven of nine participants showed a positive priming effect and were therefore included in further analyses shown below, but qualitatively similar results were obtained with all nine participants (see electronic supplementary material, figure S1).

Figure 2*b* shows the priming effect we observed in experiment 1. There was robust priming also for visible within-cue primes (i.e. when both prime and probe shapes were defined by position cues). Importantly, however, rendering the prime invisible by reversing stimulus contrast at 120 Hz revealed a dissociation. There was a strong within-cue priming effect; however, we found no cross-cue priming effect for invisible primes. In a two-way repeated-measures ANOVA with factors awareness (visible, invisible) and integration (within-cue, cross-cue), there was a significant interaction between awareness and integration (*F*_1,6_ = 15.06, *p* = 0.008), but no significant main effects of integration (*F*_1,6_ = 0.12, *p* = 0.743) or awareness (*F*_1,6_ = 4.61, *p* = 0.075).

We further explored whether the lack of cross-cue priming we observed for invisible primes was due to a slowing of response times for primed trials, or speeding up responses to unprimed trials. Figure 2*c* shows the median response times for all experimental conditions. Conscious processing of the position-defined prime afforded participants with a benefit speeding up their behavioural responses (see electronic supplementary material). Taken together, we found no cross-cue priming benefit for invisible position-defined primes. Therefore, in the absence of awareness, position cues are not integrated into a representation of global form. Finally, in an additional experiment, we confirmed that our method of rendering the primes invisible was effective (see electronic supplementary material).

### (b) Experiment 2: orientation-defined primes

Next, we investigated whether the results from the first experiment generalize to other visual features; that is, whether without awareness no spatial integration of simple visual features occurs. We used the same behavioural priming paradigm to test whether orientation cues can be grouped into a representation of global form in the absence of awareness. Participants were again asked to report whether the probe shape was a square or a diamond. However, in this experiment all primes were defined by orientation cues (figure 3*a*). As in experiment 1, accuracy in this experiment was close to ceiling levels (mean: 0.96; minimum: 0.87). Seven of nine participants showed a positive priming effect and were therefore included in further analyses shown below, but qualitatively very similar results were obtained with all nine participants (see electronic supplementary material, figure S2).

Figure 3*b* shows the priming effect observed in experiment 2. As for position-defined primes, there was robust within-cue priming by visible orientation-defined primes when the probe shape was also defined by orientation cues. Surprisingly, however, when the prime was rendered invisible, we observed *only* a strong priming effect for the cross-cue condition, that is when the probe shape was defined by position cues. There was no within-cue priming benefit. In a two-way repeated-measures ANOVA with factors awareness (visible, invisible) and integration (within-cue, cross-cue), there was a significant interaction between awareness and integration (*F*_1,6_ = 28.67, *p* = 0.002), but no significant main effects (awareness: *F*_1,6_ < 1; integration: *F*_1,6_ = 2.11, *p* = 0.197).

To explore these results further, we analysed the response times for all experimental conditions separately (see figure 3*c* and electronic supplementary material). Conscious processing of orientation-defined primes is necessary to speed up participants' responses when the probe is also defined by orientation. More importantly, however, our observation that cross-cue priming persisted when the prime is invisible strongly suggests that orientation information must somehow be integrated into a global representation even in the absence of awareness. We further confirmed that rendering the primes invisible was effective (see electronic supplementary material).

### (c) Experiment 3: retinotopic specificity of priming

The previous experiment left unclear why there should be no within-cue effect for invisible orientation-defined primes despite the fact that we observed robust cross-cue priming. If invisible orientation cues are integrated to activate a global shape representation, this should theoretically afford a priming benefit independent of the cue defining the probe shape.

However, the pattern of results we observed would also be consistent with the recruitment of lateral connections within the primary visual cortex by the invisible orientation stimuli. Such lateral connections have been suggested to play a role in contextual effects and spatial integration [14–17]. It has also been shown that, at least in tree shrews, these connections link retinotopic locations along an axis collinear with the orientation of a neuron's receptive field [18,19], which would make them an ideal candidate mechanism for activating neurons along the borders of a global shape. However, their involvement in perceptual integration remains controversial [20,21], and it is very likely that, at least for visible stimuli, recurrent feedback mechanisms from higher cortical areas into the primary visual cortex are also involved in these processes [22].

**Figure 4.**
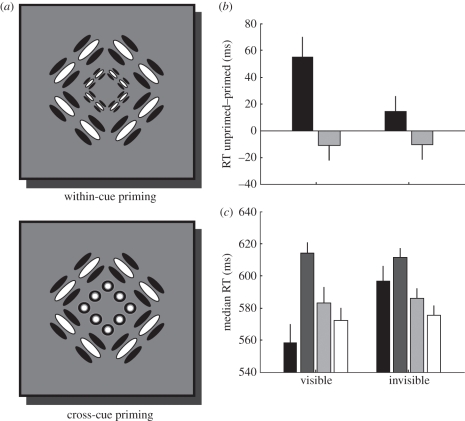
Retinotopic specificity of priming. (*a*) Illustration of prime and probe stimuli. The prime was presented at a larger scale than the probe, such that the individual elements of the probe were located outside the region where they would be expected to fall within the reach of lateral connections in visual cortex. (*b*) The priming effect. (*c*) Median response time for every trial type. All other details are identical to figure 2. (*b*) Black bars, within-cue priming; grey bars, cross-cue priming. (*c*) Within-cue: black bars, primed; dark grey bars, unprimed; cross-cue: light grey bars, primed; white bars, unprimed.

But neuronal activity in response to an oriented Gabor patch, rendered invisible as in our experiment, could spread along these connections. Perhaps this activation aided participants in detecting the presence of position cues in a probe shape in the cross-cue condition of experiment 2. This scenario makes a prediction for what happens if the probe shape was presented at a different scale than the prime. This manipulation effectively shifts the elements of the probe shape away from the retinotopic locus of the lateral connections activated by the prime stimulus (figure 4*a*). We expected to find no cross-cue effect for invisible orientation-defined primes if priming was indeed mediated by these retinotopic circuits.

As in the previous experiments, accuracy in this task was very high across all participants (mean: 0.95; minimum: 0.85). We only observed consistent within-cue priming; that is, there was a benefit for presenting an orientation-defined prime and the identical stimulus, albeit at a smaller scale (figure 4*b*). In contrast, there was no cross-cue effect, so priming by orientation cues did not transfer to probes defined by position cues. Importantly, rendering the prime invisible obliterated any priming effects. In a two-way repeated-measures ANOVA with factors awareness (visible, invisible) and integration (within-cue, cross-cue), there was a significant interaction between awareness and integration (*F*_1,6_ = 10.23, *p* = 0.024), but no significant main effects (awareness: *F*_1,6_ = 3.92, *p* = 0.104; integration: *F*_1,6_ = 4.61, *p* = 0.085).

Responses to within-cue primed trials were faster than those to unprimed trials only when the prime was visible (figure 4*c* and electronic supplementary material). Taken together, this experiment showed that rendering the prime invisible completely obliterated the priming benefit when the elements of the probe shape were now at retinotopic locations that did not fall on the borders of the global shape. Thus, the invisible priming effects we observed in the previous two experiments were probably mediated by mechanisms acting locally in retinotopic (or spatiotopic) space. Interestingly, since we also observed no cross-cue priming effect for visible primes in this experiment, even for visible stimuli the cross-cue effects may involve at least a degree of local processing.

### (d) Experiment 4: local orientation priming

Finally, we wanted to explore why no within-cue effect was observed for invisible orientation-defined primes in experiment 2. The shape-discrimination task for orientation-defined probe shapes is essentially an orientation-discrimination task. If the orientations of all the Gabor patches in the stimulus are cardinal (vertical, horizontal), the shape is a square. If they are all oblique, the shape is a diamond. Theoretically, a participant could therefore solve this task without any concept of the global shape. The invisible Gabor patches of the prime stimuli in experiment 2 were presented in the same retinotopic location as the Gabor patches in the probes. We reasoned that there should be no priming benefit by invisible oriented Gabor patches, regardless of whether the stimulus had a global shape interpretation.

**Figure 5.**
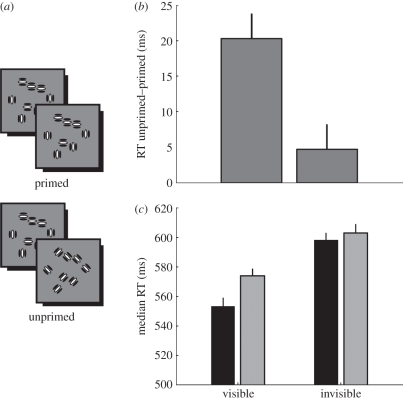
Local orientation priming. (*a*) Illustration of two trial types. The prime was always defined by orientation cues. In primed trials, the probe stimulus was the identical image. In unprimed trials, the element locations were identical as in the prime, but they were rotated by 45°. (*b*) The priming effect. (*c*) Median response time for every trial type. There was no cross-cue condition in this experiment. All other details are identical to figure 2. (*c*) Black bars, primed; grey bars, unprimed.

We tested this by modifying the procedure of experiment 2. All stimuli contained oriented Gabor patches; no position cues were used in this experiment. Instead of performing a shape-discrimination task, participants were required to judge whether the Gabor elements in the probe stimulus were all at cardinal or oblique orientations. We removed any global shape interpretation by generating stimuli consisting of eight randomly positioned Gabor patches. The orientations were consistent with those used in the previous experiments; however, because of the random placement of the elements, they could not be interpreted as shapes. The locations of the Gabor patches were randomized between trials, but within each trial they were identical in the prime and the probe (figure 5*a*).

Accuracy in this orientation-discrimination task was very high (mean: 0.94; minimum: 0.85); therefore, we concentrated our analysis on response times. There was a robust priming effect when the prime was visible. However, when the prime was rendered invisible, the priming benefit disappeared (figure 5*b*). This result replicates the within-cue effect in experiment 2. The effect of invisible primes was significantly weaker than that for visible primes (paired *t*-test: *t*_12_ = 2.2, *p* = 0.046). In the absence of awareness, presenting Gabor patches in the same retinotopic location and with identical orientation as in the probe stimulus does not afford a behavioural advantage for performing the task. Additional analysis showed that overall response times for visible trials were significantly shorter than for invisible trials (see figure 5*c* and electronic supplementary material).

## 4. Discussion

In this study, we used a priming paradigm to address two previously unresolved questions about how the brain processes information about a visual scene: First, how are discrete, local features of the input from the retina integrated into a representation about global shape? Second, in what way do these processes depend on whether we are consciously aware of the stimulus? We found that *invisible* prime shapes could exert an influence on participants' performance in a simple shape-discrimination task, as participants' response times were faster when the probe was the same shape as the prime. When the prime only contained position information, the priming benefit was observed only if the probe was the identical image (experiment 1; within-cue priming). However, when the prime was constructed of oriented elements and position information was ambiguous as to the global shape, a priming benefit was observed for probes that contained only position information. This suggested that a global representation could be inferred from the local information in the prime stimulus (experiment 2; cross-cue priming). Critically, invisible primes only exerted this effect when the prime and probe shapes were presented at the same scale, but not when the probe was smaller, which shows that the integration exploits retinotopic circuitry (experiment 3). Further, priming participants with invisible oriented elements did not provide a benefit in response times for judging the orientation of elements presented in the same spatial locations as the prime elements (experiment 4). Taken together, this pattern of results suggests that invisible oriented elements can recruit local (retinotopic) mechanisms for facilitating the detection of stimuli, but there is no integration of local information into an abstract concept of global shape.

Our findings thus reveal an important distinction between conscious and unconscious visual processing: in the absence of awareness, sparse visual stimuli constructed of a small number of local elements do not appear to activate abstract representations of geometric shape, regardless of the defining cue. While we found integration of local orientation signals into a global shape representation, this representation was not invariant to the spatial scale of the stimulus and thus was constrained to retinotopic space. This indicates that consciousness is necessary for recognizing even simple objects. Moreover, since the effects we observed for invisible primes were retinotopically constrained, our results are consistent with the notion that processing within the early visual cortex occurs regardless of awareness [4,6].

Interestingly, even for visible stimuli we found retinotopically constrained priming when the prime stimulus was defined by orientation cues, but the probe shape was defined by position cues and presented at a different scale. Only when both the prime and probe were defined by orientation was there a size-invariant benefit of being presented with the same shape. Even when participants are aware of a stimulus, retinotopic mechanisms may play an important role in activating a global shape representation. This reveals a considerable limit of the involvement of higher-order mechanisms sensitive to the abstract shape in the human visual system. While there is good evidence for the involvement of higher-level processes in perceptual integration [20–22], our findings indicate that during conscious processing integration occurs through local, retinotopic and feedback mechanisms.

How can we reconcile our findings with the observation that relatively complex stimuli, such as objects, faces and words, can have effects on behaviour and physiology in the absence of awareness? For instance, presenting observers with fearful faces that are masked from conscious awareness activates the amygdala [10]. Rendering images of faces and houses invisible through binocular fusion by presenting stimuli with opposite colour contrast to each eye nonetheless causes category-selective activations in the ventral extrastriate cortex [7,9]. Moreover, very complex and abstract information can be represented in the brain even in the absence of awareness [23].

One reason for this discrepancy may be that the neural representation of at least some complex stimuli actually differs between conscious and unconscious processing. As previous studies have shown, functional magnetic resonance imaging (fMRI) can reliably distinguish spatial patterns of neuronal activity evoked by faces and houses, regardless of whether the stimuli are visible or masked from awareness [8,9]. In these experiments, a pattern classifier is trained to discriminate the spatial activity patterns for two types of stimuli, and the trained classifier is then tested on its ability to also decode or predict the stimulus category giving rise to independent response patterns. Crucially, however, these studies both found that it was impossible to generalize between visible and invisible stimuli; that is, when the classifier was trained to discriminate visible faces and houses, it subsequently performed very poorly when used to predict the category of invisible stimuli. This indicates that the local spatial pattern of activity associated with a complex visual stimulus differs depending on whether an observer is consciously aware of it or not, and this is consistent with our present findings: without awareness, there is no abstract representation of complex visual geometry, but rather individual features are represented in isolation. They may interact through retinotopic circuits in the early visual cortex but are not bound into a global entity.

Moreover, the behavioural and physiological effects of invisible/subliminal emotional stimuli, such as images of fearful faces, are likely to involve primal mechanisms that circumvent more complex visual processing altogether [10,11,24]. Responses measured in the amygdala to invisible emotional stimuli do not arise through the geniculostriate pathway [25,26]. The response to fearful face images relies on low spatial frequency information [27]. The mechanisms for evoking emotional responses are of behaviour relevance and probably evolutionarily old. The stimuli in our study, on the other hand, comprised small local elements that can be interpreted as abstract (albeit simple) geometrical shapes. This requires higher-level conceptual processing mediated by the retinotopic cortex. Thus, our results indicate that a limit for unconscious processing may lie in the integration of limited, local information into global form.

Naturally, whenever a stimulus is manipulated in order to mask it from conscious awareness, negative findings for an effect of invisible stimuli can be attributed to the stimulus rather than to the manipulation of consciousness *per se*. In particular, with respect to our paradigm, it could be argued that the fast counter-phase flicker of the stimulus is beyond the temporal limit for producing a stimulus-associated response in the visual cortex. But we found in the first experiment that within-cue priming by position information is preserved for primes rendered invisible by this method. More importantly, in our second experiment, we also observed cross-cue priming by invisible orientation-defined primes—that is, transfer of the shape information inferred from orientation cues to shapes constructed from position cues. These findings are sufficient to demonstrate that our method of rendering stimuli invisible can still result in visual cortex responses sufficient to produce priming. This is also consistent with electrophysiological evidence demonstrating that fast-flickering stimuli entrain responses of neurons in the visual cortex [28–30]. Moreover, recent behavioural experiments showed that stimuli rendered invisible by this method nonetheless produce adaptation to oriented gratings that are consistent with a neural representation in the primary visual cortex [31]. Taken together, the absence of priming effects we found in our study must have resulted from the failure of the stimulus to reach consciousness, rather than it failing to produce reliable visual responses.

What could be the neural substrates underpinning our unconscious priming effects? Our unconsciously presented prime stimuli must leave a trace in the retinotopic space occupied by the global shape, which draws the observer's attention to these locations and thus facilitates the detection and discrimination of subsequently presented probe stimuli. In the case of within-cue priming by position cues (experiment 1), participants are already primed to the locations of the probe elements allowing them to respond more quickly than if the opposite shape was presented. In contrast, when priming participants with invisible orientation-defined shapes, locations different from those of the prime elements—but which were collinear with the sides of the probe shape—were also primed, affording a behavioural facilitation for detecting position-defined probes (experiment 2).

A likely candidate circuit to mediate such effects could be the long horizontal connections between neurons in the primary visual cortex [14,15,17]. They have been reported to connect neurons with similar orientation tuning across several degrees in visual space [18,19,32,33], and have been suggested to play a role in various forms of spatial integration [34,35]. While their involvement in contour integration remains controversial [20,21], it can explain the effects we observed here. Experiments in tree shrews showed that the spread of these connections from a neuron is not unidirectional but tends to favour neurons that lie on a collinear axis in visual space with the neuron's preferred orientation [18,19], and that these connections explain the facilitation of neuronal responses to collinear grating stimuli. More recently, voltage-sensitive dye imaging in behaving monkeys demonstrated that small grating stimuli produce ‘filling-in’ of surrounding regions, but more readily in regions that are collinear with the stimulus orientation than with orthogonal ones [36]. Further, there is evidence that lateral interactions in the visual cortex are stronger for low-contrast stimuli, suggesting more reciprocal facilitation [37]. Importantly, all the stimuli in our current study were at low (but supra-threshold) contrast levels, which further suggests a role for these connections in our experiment. Future neurophysiological studies should explore in how far the fast counter-phase flicker we employed here affects the activation of horizontal connections in the visual cortex.

Finally, we also found a lack of within-cue priming for orientation-defined primes that had been rendered invisible. At first glance, this result may seem counterintuitive, because the presence of invisible cross-cue priming from orientation- to position-defined shapes would suggest that within-cue priming (that is, priming by an *identical image*) should also be observed. However, we believe that this finding is in fact consistent with our main hypothesis. Judging the shape of our orientation-defined probes in the first three experiments is an implicit orientation-discrimination task. Moreover, in our fourth experiment, participants were explicitly instructed to discriminate the orientation of randomly positioned elements without any global shape interpretation. These experiments demonstrated that invisible oriented elements do not afford any behavioural advantage for discriminating the orientations in the probe stimulus. This is also consistent with reports of adaptation to oriented grating stimuli rendered invisible using the same method [31]. It is possible that the invisibly presented oriented elements reduce the contrast sensitivity for discriminating the subsequently presented low-contrast orientation elements in the probe stimulus. But because the facilitation we observed for visible primes appears to occur through speeding up the responses in primed trials, we believe a more parsimonious explanation is that what must have been primed is an abstract representation of the stimulus orientation, which may be of a semantic or at least conceptual nature. Our results suggest that conscious processing of orientation is therefore necessary to activate this representation.

## 5. Conclusions

In this study, we explored the extent to which invisible stimuli can prime behavioural performance for discriminating geometrical shapes. We found that while position information is not integrated into a representation of global form in the absence of awareness, orientation information is. However, our results suggest that this spatial integration is retinotopic, which implicates the involvement of lateral horizontal connections in the primary visual cortex being involved in this process. Our results contribute to our understanding of visual processing by demonstrating that such local mechanisms play an important role in spatial integration. Importantly, we show that when stimuli are masked from awareness, this circuitry can mediate spatial integration and exert an influence on behaviour; critically, however, an abstract representation of even primitive geometric information depends on consciousness.
